# Evaluating socioeconomic inequalities in self-rated oral health and its contributing factors in Brazilian older adults

**DOI:** 10.1371/journal.pone.0316145

**Published:** 2025-01-03

**Authors:** Fabíola Bof de Andrade, José Leopoldo Ferreira Antunes

**Affiliations:** 1 Oswaldo Cruz Foundation, Rene Rachou Institute, Belo Horizonte, Brazil; 2 Department of Epidemiology, School of Public Health, University of São Paulo, São Paulo, Brazil; Kuwait University, Faculty of Dentistry, KUWAIT

## Abstract

**Objectives:**

This study aimed to evaluate socioeconomic inequalities in self-reported oral health among community-dwelling Brazilian older adults and evaluate the oral health factors contributing to the inequalities.

**Methods:**

This was a cross-sectional study with data from the Brazilian National Health Survey conducted in 2019. The dependent variable is the self-report of oral health categorized as good or poor. Household per capita income in quintiles and schooling were used as socioeconomic variables. The explanatory covariates were age; gender; limitation in basic activities of daily living; number of teeth, use of dental prostheses; difficulty in eating; and recent dental visit. The Oaxaca-Blinder two-fold decomposition for binary outcomes was used to evaluate the factors contributing to the inequalities in self-reported oral health.

**Results:**

Self-reported poor oral health was found among 35.8% of the dentate and 29.6% of the edentulous individuals. Poor self-reported oral health was more prevalent among older adults with low income and educational levels. Among dentate individuals, the difference in the proportion of poor self-reported oral health (the gap) between those with no schooling and those with some schooling was 12.8 percent points (p.p.), favoring the poor. The gap between dentate in the lowest and highest income groups was 14.8 p.p. favoring the poor. Among edentulous individuals, those with no schooling had a higher proportion of self-reported oral health (total gap 10.6 p.p.). Concerning income inequalities, the gap favored the poorer group and was 5.4 p.p. higher among individuals in the lowest income group.

**Conclusion:**

The decomposition analyses suggested that oral health variables explained most of the education and income inequalities; difficulties in eating were the most contributing factor in both the dentate and edentulous groups. There was a relatively reduced contribution of recent dental visits to socioeconomic inequality.

## Introduction

Oral health is an integral component of overall well-being, encompassing physical, psychological, and social dimensions that collectively contribute to an individual’s quality of life [1]. In recent years, increasing attention has been directed towards quantifying the magnitude of oral health socioeconomic inequalities, particularly those related to clinical oral health measures [2, 3] and access to dental care services [4]. It is well acknowledged that individuals from lower socioeconomic strata experience a higher burden of oral health problems [2, 3] or have lower access to oral health care [4].

Moreover, a growing body of evidence underscores the need for a comprehensive analysis that establishes the association between socioeconomic position measures, such as education and income and oral health, and disentangles the contributing factors through advanced analytical techniques [5–8]. Understanding the composition of the health gap and its contributing factors is essential for identifying the drivers of health inequalities and is crucial for developing policies to tackle them [8].

One critical yet relatively underexplored facet is the socioeconomic inequalities related to self-reported oral health. Self-reported oral health, a subjective assessment of one’s oral well-being, emerges as a crucial indicator reflecting both the objective state of oral health and the broader context in which it exists [9]. It encompasses the presence of oral diseases and subjective perceptions of pain, discomfort, and aesthetics, thus capturing the psychosocial dimensions of oral health [9, 10] and influencing dental care utilization [11]. Although some studies have investigated differences between socioeconomic groups in self-rated oral health [12–14], few to no studies have quantified its inequalities using complex measures nor investigated the contribution factors of these differences [15, 16].

The Oaxaca-Blinder technique allows for quantifying the contribution of different factors to disparities [17]. By applying this technique to study oral health socioeconomic inequalities, we can gain deeper insights into how education and income differentials influence oral health outcomes. It is worth noting that despite its potential, this method has not been extensively explored in the field of oral health. In Brazil, previous applications of Oaxaca-blinder decomposition have examined income [18] and racial inequities in tooth loss [6].

This paper aims to contribute to the existing literature by evaluating socioeconomic inequalities in self-reported oral health among community-dwelling older adults and exploring the oral health factors contributing to the inequalities.

## Methods

This study was a cross-sectional assessment of data from the Brazilian National Health Survey conducted in 2019 by the Brazilian Institute of Geography and Statistics (IBGE) in partnership with the Oswaldo Cruz Foundation (Fiocruz). This survey represents Brazilian people aged 15 or older who reside in permanent private dwellings. The sampling process included clustering in three stages. The primary sample unit was the census tract or sets of tracts, followed by households within each tract and a random selection of one resident in each household. Information regarding the sampling and weighting process was previously reported [19].

This present study uses data from individuals aged 60 and older with complete information for all variables of interest.

The Brazilian National Health Survey was submitted to the Brazilian National Research Ethics Committee/National Health Council and approved on August 23rd, 2019 (protocol number 3,529,376). Written informed consent was obtained for all the participants. All the data from the survey are publicly available at https://www.ibge.gov.br/estatisticas/sociais/saude/9160-pesquisa-nacional-de-saude.html?=&t=microdados. A dataset containing the variables used in the present analyses is available as a supplementary file.

The dependent variable is the self-report of oral health measured using the question: “How would you rate your oral health (teeth and gums)?” Five possible answers were categorized as good (very good/good) and poor (regular, poor and very poor) [12, 20].

Socioeconomic status was classified by household per capita income in quintiles and schooling. Income was categorized as low (1^st^ quintile) and higher (≥2^nd^ quintile), and schooling as low (no schooling) and higher (primary education or more).

The explanatory covariates were age (years); gender (male, female); limitation in basic activities of daily living—BADL (no, yes); oral health conditions [number of teeth, use of dental prostheses (no, yes); difficult in eating (no, yes); last dental visit (>2 years, ≤2 years)].

Difficulty in eating was evaluated using the question: “How much difficulty do you have eating because of problems with your teeth or dentures?” with five possible answers that were categorized as low (no difficult, low, moderate) and high (high, very high). Individuals reporting any limitation to perform one or more BADLs (feeding, bathing, using the toilet, dressing, crossing a room on the same floor, and getting out of bed) were classified as having limitations.

Statistical analyses included the description of the study’s variables, followed by the description of the outcome prevalence as stratified by education and income. The Oaxaca-Blinder two-fold decomposition for binary outcomes evaluated the factors contributing to the inequalities in self-reported oral health. This contrafactual analysis explains the gap in the outcome variable between two sociodemographic groups (e.g., between low-income people and the nonpoor). This method decomposes the gap (i.e., the observed disparity) into two components; the explained (attributed to observable characteristics of the groups) and the unexplained (those that these characteristics cannot explain). Using this method, we also quantified how much of the difference in the outcome variable is due to differences in the coefficients of the independent variables between the two groups of the socioeconomic stratifiers [17, 21]. The higher socioeconomic groups were used as the reference groups for the decomposition analyses. For each explanatory factor, the contributing coefficients were reported [22] and the relative contributions to the total gap (percentage of the total difference between groups [gap]). All analyses used Stata 18.0 (Stata Corp LP) and considered the complex sampling design and sampling weights.

## Results

The mean age among dentate was 68.2 years and 72.7 among edentulous individuals. In both samples, most individuals were female. About 11.5% and 17.9% of dentate individuals had no schooling and low income, respectively. These proportions corresponded to 25.9% and 23.9% among edentulous. Most older adults used dental prostheses in both samples. Self-reported poor oral health was found among 35.8% of the dentate and 29.6% of the edentulous (Table 1).

**Table 1:** Descriptive statistics.

	Dentate (14,367)	Edentulous (8,358)
	% (95% CI)	% (95% CI)
**Age (mean)**	68.2 (68.0; 68.4)	72.7 (72.4; 73.0)
**Gender**		
Female	51.7 (50.4; 53.1)	65.2 (63.5; 66.8)
**Education**		
No schooling	11.5 (10.7; 12.4)	25.9 (24.3 27.6)
**Income**		
Low	17.9 (16.9; 18.9)	23.6 (22.0; 25.3)
**BADL limitation**		
Yes	16.7 (15.7; 17.7)	27.4 (25.7; 29.1)
**Recent dental visit**		
Yes	61.0 (59.6; 62.5)	24.0 (22.4; 25.7
**Difficult to eat**		
Yes	17.8 (16.8; 18.9)	25.1 (23.5; 26.7)
**Use of dental prostheses**		
Yes	62.3 (61.0; 63.6)	88.5 (87.2; 89.6)
**Number of teeth (mean)**	19.9 (19.6; 20.2)	-
**Self-reported oral health**		
Poor	35.8 (34.4; 37.1)	29.6 (28.0; 31.3)

The S1 Table shows the distribution of the study’s variables by the socioeconomic position measures. The dentate and edentate samples’ mean age was higher among individuals with some education and higher income. Among dentate older adults, there was a lower prevalence of BADL limitation among individuals with some education (15.5%) than the ones with no schooling (25.3%). Eating difficulty was higher among those with no schooling (29.7% vs. 1.6.3% among those with some education). About 47.1% of those with no schooling had poor self-reported oral health, whereas the percentage among individuals with some education was 34.3%. Similar patterns were found for the distribution of the variables and income in the dentate group.

Concerning edentulous individuals, individuals with no schooling and the lowest income had the worst indicators of oral health and general health. Eating difficulties were reported by 33.1% of older adults with no schooling and by 22.2% of those with some schooling. Among those in the lowest income category and highest, respectively, the prevalence was 28.5% and 24.0%. Older adults with higher socioeconomic positions (education and income) had a higher percentage of recent dental visits and self-reported poor oral health. The higher schooling and income groups had, respectively a 26.9% and 28.3% frequency of self-reported poor oral health. Among those with some schooling and in the lowest income group, the distribution was 37.5% and 33.7%, respectively (S1 Table).

According to the decomposition analyses (Table 2), among dentate individuals, the difference in the proportion of poor self-reported oral health (the gap) between those with no schooling and those with some schooling was 12.8 percent points (p.p.), favoring the poor. About 87.5% (0.112/0.128) of this gap was explained by the covariates in the model. The gap between dentate in the lowest and highest income groups was 14.8 p.p. favoring the poor. The explained gap by the covariates according to income was 0.09 (60.8% of the total gap). The self-reported difficulty in eating made the highest contribution to the total gap for dentate individuals either for education (35.2%) or income inequalities (25.0%).

**Table 2:** Decomposition results of the “explained” gap for education and income-related inequality in self-reported poor oral health.

	Education				Income			
	Dentate		Edentate		Dentate		Edentate	
	Coef. (95% CI)^†^	% tot gap	Coef. (95% CI)^†^	% tot. gap	Coef. (95% CI)^†^	% tot. gap	Coef. (95% CI)^†^	% tot. gap
**Group = no education/low income**	0.471 (0.438; 0.504)		0.375 (0.341; 0.409)		0.479 (0.450; 0.508)		0.337 (0.302; 0.372)	
**Group = some education/higher income**	0.343 (0.329; 0.357)		0.269 (0.251; 0.287)		0.331 (0.317; 0.346)		0.283 (0.265; 0.301)	
Difference (total gap)*	0.128 (0.093; 0.163)		0.106 (0.068; 0.145)		0.148 (0.115; 0.180)		0.054 (0.015; 0.093)	
**Explained gap**	0.112 (0.092; 0.132)		0.070 (0.051; 0.090)		0.090 (0.074; 0.105)		0.040 (0.024; 0.057)	
**Unexplained gap**	0.016 (-0.018; 0.051)		0.036 (-0.004; 0.076)		0.058 (0.026; 0.090)		0.014 (-0.025; 0.052)	
**Age**	-0.010 (-0.016; -0.003)	-7.8	-0.002 (-0.011; 0.007)	-1.9	0.005 (0.001; 0.008)	3.4	0.002 (-0.002; 0.007)	3.7
**Female**	0.003 (0.000; 0.005)	2.3	-0.001 (-0.003; 0.002)	-0.9	0.000 (-0.002; 0.002)	0.0	-0.001 (-0.004; 0.002)	-1.9
**High income**	0.012 (0.004; 0.019)	9.4	-0.000 (-0.008; 0.007)	0.0				
**Some education**					0.001 (-0.005; 0.007)	0.7	0.003 (-0.003; 0.010)	5.6
**BADL limitation**	0.008 (0.003; 0.012)	6.3	0.006 (0.001; 0.011)	5.7	0.005 (0.002; 0.008)	3.4	0.005 (0.000; 0.009)	9.3
**Recent dental visit**	0.026 (0.016; 0.036)	20.3	0.004 (-0.001; 0.009)	3.8	0.023 (0.015; 0.031)	15.5	0.002 (-0.001; 0.006)	3.7
**Difficult to eat**	0.045 (0.032; 0.057)	35.2	0.036 (0.024; 0.048)	34.0	0.037 (0.027; 0.048)	25.0	0.015 (0.002; 0.027)	27.8
**Use of dental prosthses**	0.012 (0.003; 0.020)	9.4	0.028 (0.016; 0.040)	26.4	0.007 (0.001; 0.013)	4.7	0.014 (0.007; 0.021)	25.9
**Number of teeth**	0.017 (0.010; 0.024)	13.3			0.011 (0.006; 0.017)	7.4		

*Comparing individuals with no education versus some education or those with low income (1^st^ quintile of per capita family income) versus high income (2^nd^ quintile or more)

Among edentulous individuals, those with no schooling had a higher proportion of self-reported oral health (total gap 10.6 p.p.), and 66.0% of the gap was explained by the covariates (0.07/0.106). Concerning income inequalities, the gap favored the poorer group and was 5.4 p.p. higher among individuals in the lowest income group. The explained gap accounted for 74.1% of the total gap related to income inequalities (0.04/0.054). Similar explanatory factors were found for income and education inequalities among edentulous individuals. All explanatory variables positively contributed, except by age, in the education-inequalities decomposition. Among the oral health measures, recent dental visits made the lowest contribution to education (3.8%) and income inequalities (3.7%) in the edentulous group (Table 2).

**Fig 1:**
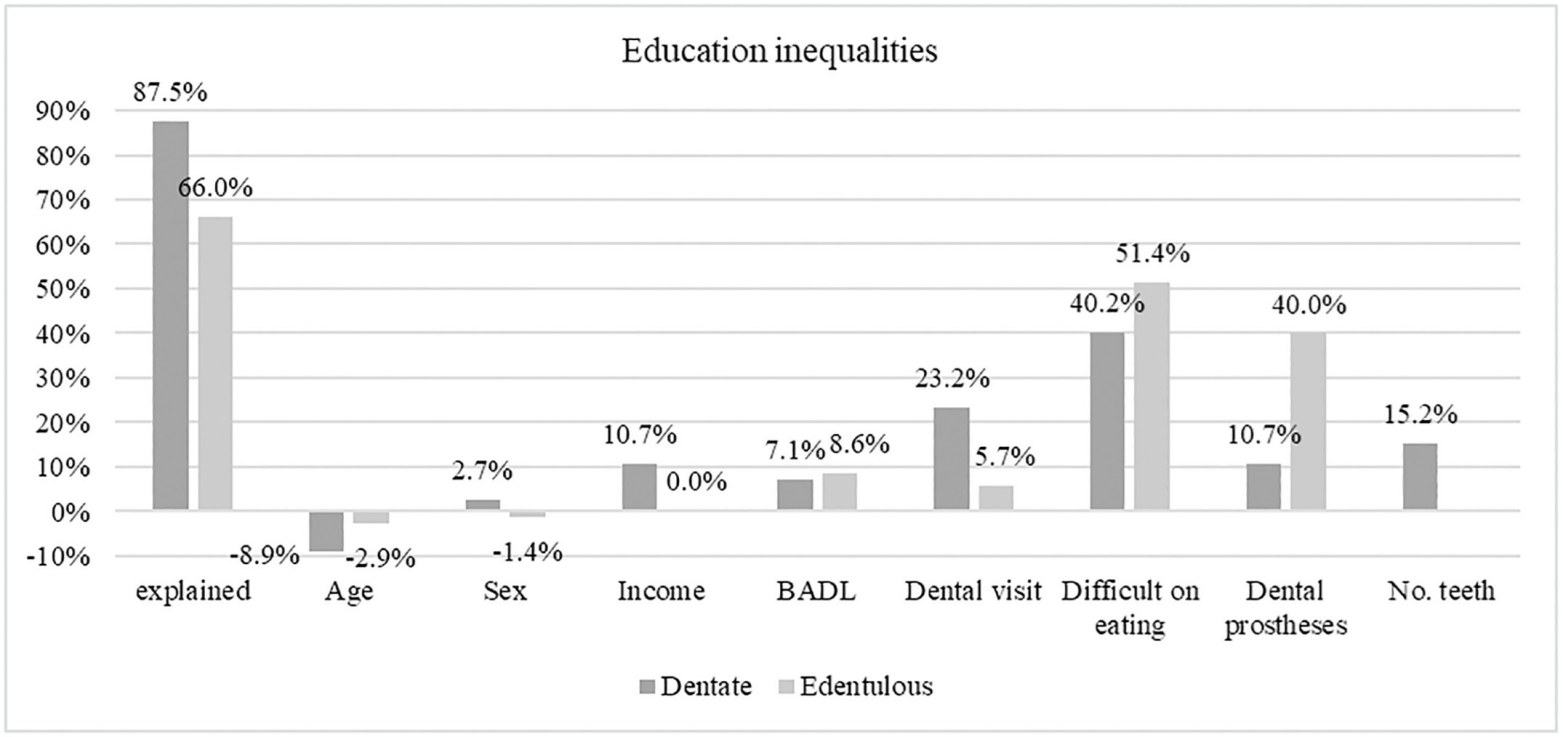
Percentage contributions of each factor to the explained gap in the prevalence of poor self-reported oral health between individuals with no education versus some education.

**Fig 2:**
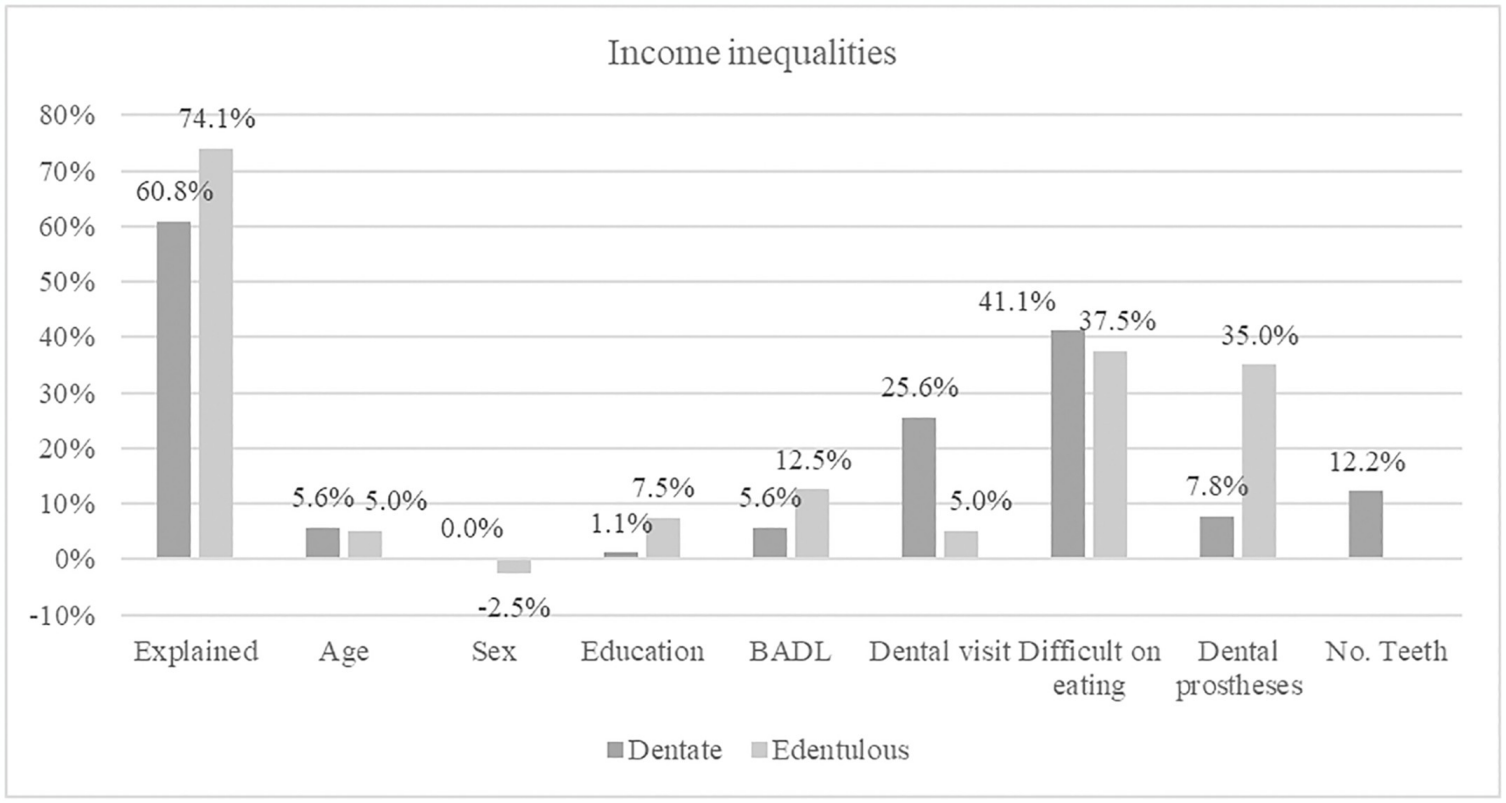
Percentage contributions of each factor to the explained gap in the prevalence of poor self-reported oral health between individuals with low income (1^st^ quintile of per capita family income) versus high-income.

Figs 1 and 2 show the percentage of each explanatory variable’s contributions to the explained gap of education and income inequalities, respectively. The explained contribution was lower for the edentulous group concerning education inequalities, whilethe opposite was found for income inequalities. Self-reported difficulty in eating made the highest contribution to the explained gap for dentate (education 40.2%; income 41.1%) and edentulous (education 51.4%; income 37.5%). The positive coefficient suggests that this factor contributes to the increase of inequalities, meaning that if individuals with no schooling (or low income) had the same level of perceived eating ability as those with some education (or higher income), the proportion of self-reported poor oral health among them would be lower and the gap reduced. The use of dental prostheses, followed by the number of teeth, were the oral health measures that made the lowest contributions among dentate individuals for both education and income inequalities. Among edentulous, for both inequalities, recent dental visits followed by dental prostheses were the lowest contributors among the oral health measures.

## Discussion

This study showed that poor self-reported oral health is disproportionally concentrated among older adults with low income and educational levels. The decomposition analyses suggested that oral health variables explained most of the education and income inequalities; difficulties in eating were the most contributing factor in both the dentate and edentulous groups.

Differences in the magnitude of inequalities by socioeconomic variables and the proportion explained by each stratifier [8, 23] allign with existing evidence in the broader field of health inequalities, supporting the importance of evaluating different socioeconomic measures [24–26]. The choice of the socioeconomic classification should consider the health outcome and contextual factors. However, variables of economic status, such as wealth and income, have been the mostly used [25, 27]. Two previous studies that evaluated self-reported oral health among adults found that the magnitude of relative income inequalities, assessed by the relative index of inequality, was more significant than education [24, 28]. Similar findings have been found for absolute inequalities, measured using the slope index of inequalities [28]. Unlike this, no specific pattern was observed in the present study. The point estimate for the gap was higher for income inequalities among dentate and for education inequalities for edentulous individuals. Income and education are proxies for a complex interplay of factors, including access to care, health behaviors, psychosocial factors, and early life influences. This study’s insights into the differential impacts of income and education on dentate and edentulous populations highlight the need for tailored public health interventions that address the specific needs of these groups [29, 30].

This study presents significant insights into the role of dental care services in mitigating oral health inequalities. While enhancing access to dental care has been linked to reducing socioeconomic inequalities in oral health, [31] it is critical to note that its impact on self-reported general health [8, 23] and oral health [16, 32] is smaller than the influence of other factors, corroborating the current findings. Within the scarce evidence based on decomposition analysis, a population-based study with adults from Thailand found that publicly-subsidized insurance and dental care utilization contributed 2,4% and 15.4%, respectively, for the self-reported poor oral health inequality (evaluated by the concentration index) [16]. This contribution is similar to the one found for the use of dental care in the current study.

Although the covariates explained 60% or more of the gaps in all models, a significant portion of socioeconomic differences in poor self-reported oral health remained unexplained. The unexplained gap reflects differences in how the predictor variables relate to the outcome across socioeconomic groups. This suggests that the same level of a predictor may result in different health outcomes depending on the social group. Another potential reason for the unexplained gap is the presence of unobserved factors (e.g., quality of education, differences in healthcare provision, social and cultural values, health-related beliefs) that were not included in the model [21, 23, 33]. Therefore, future studies should explore additional explanatory variables not evaluated in the present study.

The low contribution of dental care to reduce oral health inequalities may rely on the frequency and reasons for seeking dental care among this age group. Moreover, the differences in the use and provision of services should be considered. Social and cultural values, health-related beliefs, and behaviors, besides costs, are associated with the use of dental care and can mediate the effect of socioeconomic positions on this outcome [34, 35]. Although the Brazilian Public Health System has a large basket of dental care services, free of charge at the point of care, there is a low prevalence of use among older adults, almost exclusively for treating dental symptoms rather than for regular check-ups [36].

This context also explains the even lower contribution of services to edentulous individuals and the proportion explained by the number of teeth since the prevalence of severe tooth loss is high. In Brazil, among adults 20 years or older from São Paulo, not having teeth and financial difficulty were the most common reasons for not seeking dental care in lower socioeconomic groups. Deeming that dental services were unnecessary was more common among higher socioeconomic groups [37]. This is in line with the findings from a study conducted with older adults 50 years and older from 13 European countries. It was found that perceiving regular dental treatment as ‘not necessary’ or ‘not usual’ was the predominant reason for non-attendance in all welfare state regimes [38]. In addition, it is worth noting that the lack of use for check-up purposes limits the dental care services’ ability to act in a preventive mood.

While utilization of dental care showed a negligible impact on mitigating oral health inequalities, the contrasting finding of a higher explanatory proportion related to clinical oral health measures, notably difficulties in eating, sheds light on the potential transformative contribution of dental care services if tailored to address the population needs. The use of dental prostheses and the report of difficulties in eating are both measures of functionality with a psychosocial dimension, including dental appearance, in the case of the former. Functional aspects of oral health have been increasingly recognized as essential components in understanding overall well-being [1]. Their high contributions to the total gap underscore that rehabilitation services are not universally accessible. Indeed, dental prostheses are available in the Brazilian Public Health System. However, this service is subject to many barriers to access and provision, [39, 40] being mostly implemented by the private health sector [41].

The primary strength of this study lies in its comprehensive analysis of contributing factors to oral health inequalities using advanced analytical techniques like the Oaxaca-Blinder decomposition. This approach allows for a more in-depth understanding of how specific factors contribute to inequality. Among the study’s limitations, the cross-sectional design restricts the ability to draw causal inferences. The reliance on self-reported oral health measurements, while valuable for understanding perceived oral health status, should also be recognized. In addition, it is worth noting that factors other than actual oral health status and general health conditions, such as personal preferences and awareness could contribute to the inequalities and for improving the explained part of the gap. Findings reported here are specific to the context of the Brazilian older adults which might limit the generalizability to other regions with different cultural, economic, and healthcare contexts.

Our study showed significant inequalities in self-reported oral health based on socioeconomic factors, such as income and education. The findings underscore the role of functional aspects of oral health in understanding oral health inequalities. The relatively limited contribution of recent dental visits to these inequalities suggests the need for further investigation into systemic barriers that may hinder access to rehabilitative dental services and the role of such care in improving oral health. Given the higher prevalence of poor self-reported oral health among those with lower income and education levels and the significant impact of functional difficulties, public policy needs to understand if these services, offered free of charge at the point of care through the public health system, are underutilized or not effective in addressing these group needs. Educational initiatives to enhance health literacy among older adults in disadvantaged socioeconomic groups are crucial for preventing oral diseases. Finally, integrating oral health into comprehensive geriatric care models can help reduce these disparities and improve the overall quality of life for older adults.

## Supporting information

S1 Table Distribution of variables by education and per capita family income among dentate and edentulous older adults. (DOCX)

S1 Data (CSV)
