# Supplementary material for: Evaluating socioeconomic inequalities in self-rated oral health and its contributing factors in Brazilian older adults
Source: PLoS One. 2025 Jan 3;20(1):e0316145. doi: 10.1371/journal.pone.0316145 (PMC11698338; doi:10.1371/journal.pone.0316145)
Supplement: S1 Table — (DOCX) [file pone.0316145.s001.docx]

S1 Table – Distribution of variables by education and per capita family income among dentate and edentulous older adults

|  | **Education** | | **Income** | |
| --- | --- | --- | --- | --- |
|  | **No** | **Some** | **Lowest (1^st^ quintile)** | **≥2^nd^ quintile** |
| **DENTATE** | **%** |  | **%** |  |
| **Age (mean)** | 71.0 (70.4; 71.6) | 67.8 (67.6; 68.0)*** | 66.8 (66.5; 67.2) | 68.5 (68.3; 68.7)*** |
| **Gender** |  | * |  |  |
| Female | 47.7 (44.0; 51.4) | 52.2 (50.8; 53.6) | 51.7 (8.7; 54.7) | 51.7 (50.2; 53.2) |
| **BADL limitation** |  | *** |  | *** |
| Yes | 25.3 (22.4; 28.5) | 15.5 (14.5; 16.6) | 22.2 (19.8; 24.7) | 15.5 (14.5; 16.6) |
| **Recent dental visit** |  | *** |  | *** |
| Yes | 33.1 (29.7; 36.7) | 64.7 (63.2; 66.2) | 41.7 (38.8; 44.7) | 65.3 (63.7; 66.8) |
| **Eating difficult** |  | *** |  | *** |
| Yes | 29.7 (26.5; 33.2) | 16.3 (15.2; 17.4) | 26.7 (24.2; 29.5) | 15.9 (14.8; 17.0) |
| **Use of dental prostheses** |  | *** |  | *** |
| Yes | 41.3 (37.7; 45.1) | 65.1 (63.7; 66.5) | 49.1 (46.1; 52.0) | 65.2 (63.8; 66.7) |
| **Number of teeth (mean)** | 17.1 (16.5; 17.8) | 20.3 (20.0; 20.6)*** | 18.2 (17.6 18.7) | 20.3 (20.0; 20.6)*** |
| **Self-reported oral health** |  | *** |  | *** |
| Poor | 47.1 (43.4; 50.8) | 34.3 (32.9; 35.7)*** | 47.9 (45.0; 50.8) | 33.1 (31.6; 34.6) |
| **EDENTULOUS** |  |  |  |  |
| **Age (mean)** | 75.3 (74.7; 76.0) | 71.8 (71.4; 72.2)*** | 71.2 (70.5; 71.9) | 73.2 (72.8; 73.6)*** |
| **Gender** |  |  |  |  |
| Female | 66.0 (63.1; 68.9) | 64.8 (62.8; 66.8) | 66.3 (62.7; 69.8) | 64.8 (62.9; 66.6) |
| **BADL limitation** |  | *** |  | * |
| Yes | 34.0 (30.8; 37.3) | 25.0 (23.1; 27.1) | 31.3 (27.5; 35.3) | 26.1 (24.3; 28.0) |
| **Recent dental visit** |  | *** |  | ** |
| Yes | 16.1 (13.5; 19.0) | 26.8 (24.8; 28.8]) | 18.4 (15.7; 21.5) | 25.7 (23.9; 27.7) |
| **Eating difficult** |  | *** |  |  |
| Yes | 33.1 (30.1; 36.3) | 22.2 (20.4; 24.1) | 28.5 (25.2; 32.0) | 24.0 (22.2; 25.9) |
| **Use of dental prostheses** |  | *** |  | *** |
| Yes | 76.9 (73.7; 79.8) | 92.5 (91.3; 93.6) | 82.0 (78.5; 84.9) | 90.5 (89.3; 91.6) |
| **Self-reported oral health** |  | *** |  | ** |
| Poor | 37.5 (34.1; 41.0) | 26.9 (25.1; 28.7) | 33.7 (30.3; 37.4) | 28.3 (26.5; 30.3) |
| *p<0.05; **p<0.01; p<0.001 |  |  |  |  |
